# Macular telangiectasia with bilateral obliterated capillaries: a case report

**DOI:** 10.1186/s13256-022-03597-z

**Published:** 2022-10-13

**Authors:** Melika Samadi, Afsaneh Azarkish, Hamid Riazi-Esfahani, Alireza Mahmoudi, Nazanin Ebrahimiadib

**Affiliations:** 1 Retina Service, Farabi Eye Hospital, Tehran University of Medical Sciences, Tehran, Iran; 2 Department of Ophthalmology, School of Medicine, Dezful University of Medical Sciences, Dezful, Iran

**Keywords:** Macular telangiectasia, MacTel type 3, MacTel classification

## Abstract

**Background:**

We report an otherwise healthy adult with macular telangiectasia with aneurysms, ischemia, and obliterated capillaries in both eyes.

**Methods:**

This is a case report with a brief literature review.

**Case presentation:**

A 58-year-old Iranian woman presented with a gradual decrease in vision, with recent deterioration. Past medical history was unremarkable, and best-corrected visual acuity was 3/10 in both eyes. Multimodal imaging including fundus photo, fluorescein angiography, optical coherence tomography, and optical coherence tomography angiography was carried out. In macula of both eyes, parafoveal telangiectasia, occluded vessels, capillary dropouts, and aneurysms were observed. While there were a dense circinate exudation and edema in the macula of the right eye and a thin and disorganized inner retinal layer in the left eye, the outer retina was intact in both eyes. En face optical coherence tomography angiography revealed capillary blunting and rarefaction in both superficial and deep capillary plexuses of retina.

**Conclusion:**

Our case most probably represents a case of type 3 macular telangiectasia in the absence of any systemic association.

## Background

The term “macular telangiectasia” refers to the dilation of the capillary network at the parafoveal region due to congenital or developmental etiologies. Idiopathic macular telangiectasia (IMT or MacTel), first described by Gass and Oyakawa, as incompetent telangiectatic capillaries supplying the juxtafoveal area [1], was later classified by Gass and Blodi in 1993 into three types. MacTel type 1 is a developmental condition that is typically characterized by unilateral aneurysms and telangiectasia located in the temporal half of the macula, accompanied by exudation and cystoid macular edema (CME). MacTel type 2 is an acquired condition and is typically bilateral, and nonexudative and aneurysmal changes are not apparent. Among its distinctive features are superficial crystalline deposits, right-angle venules, and intraretinal pigment migration. The third type is an acquired bilateral occlusive macular telangiectasia with progressive vision loss. It has been poorly understood, and all the reported cases were associated with a systemic vascular or cerebral disease.

In Gass series, type 3 was described in 7 of 140 (5%) patients [2]. Yannuzi simplified this classification in 2006, and called type 1 as aneurysmal telangiectasia and type 2 as perifoveal telangiectasia. Features of the third type were not observed, so it has been omitted due to its rarity [3]. Maruko *et al.*, in a case series of macular telangiectasia in Japanese population including 27 patients, reported four eyes of two patients (7.4%) with MacTel type 3 [4].

Here, we report an otherwise healthy woman whose clinical picture is mostly compatible with bilateral idiopathic occlusive macular telangiectasia.

## Case presentation

**Fig. 1:**
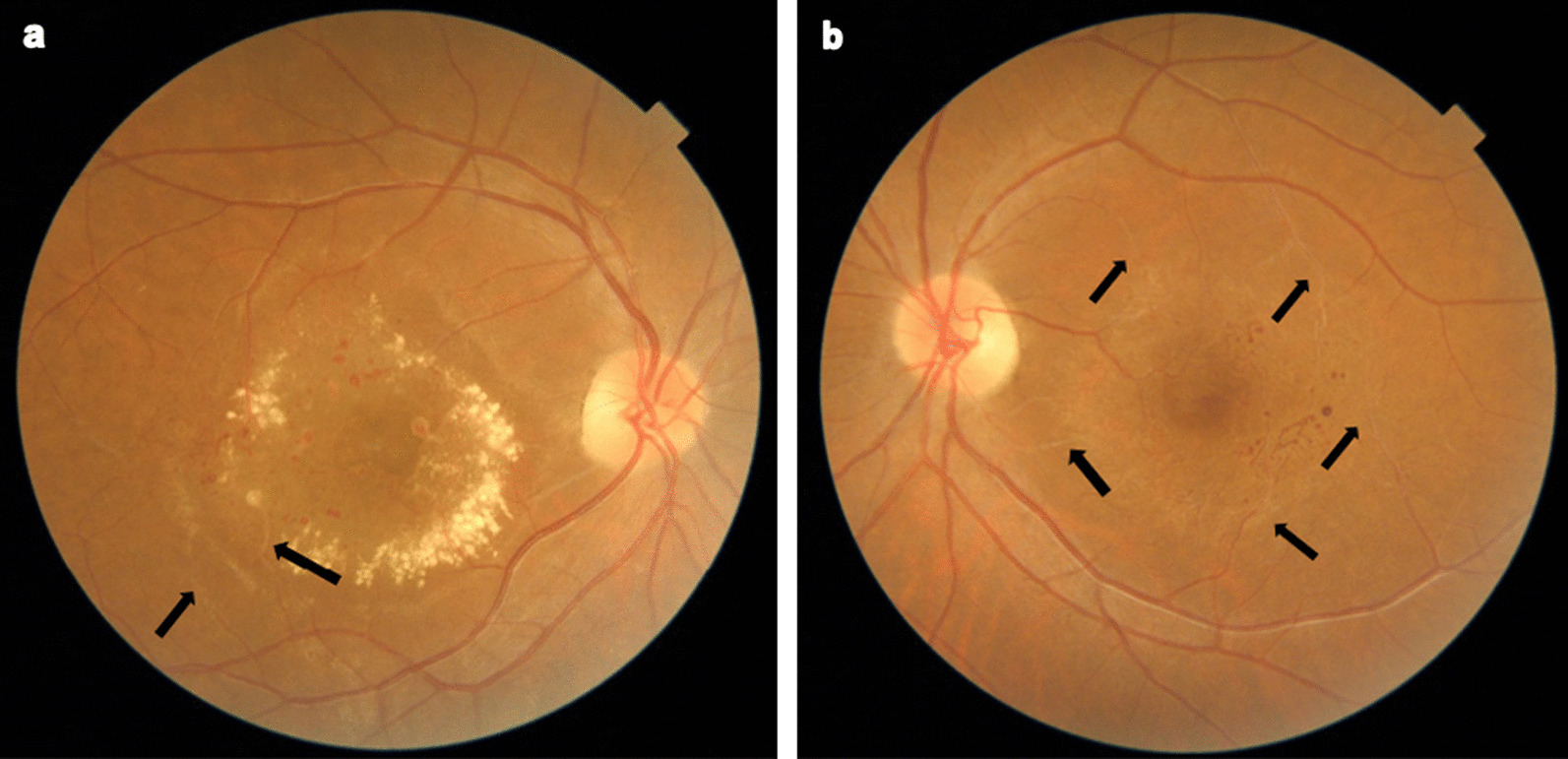
Color fundus photographs of the right (**a**) and left (**b**) eye demonstrate parafoveal telangiectatic vessels and round red lesions representing microaneurysms as well as occluded sclerotic vessels (black arrows) in both eyes. Macular edema and circinate exudation are evident in the right eye (**a**)

**Fig. 2:**
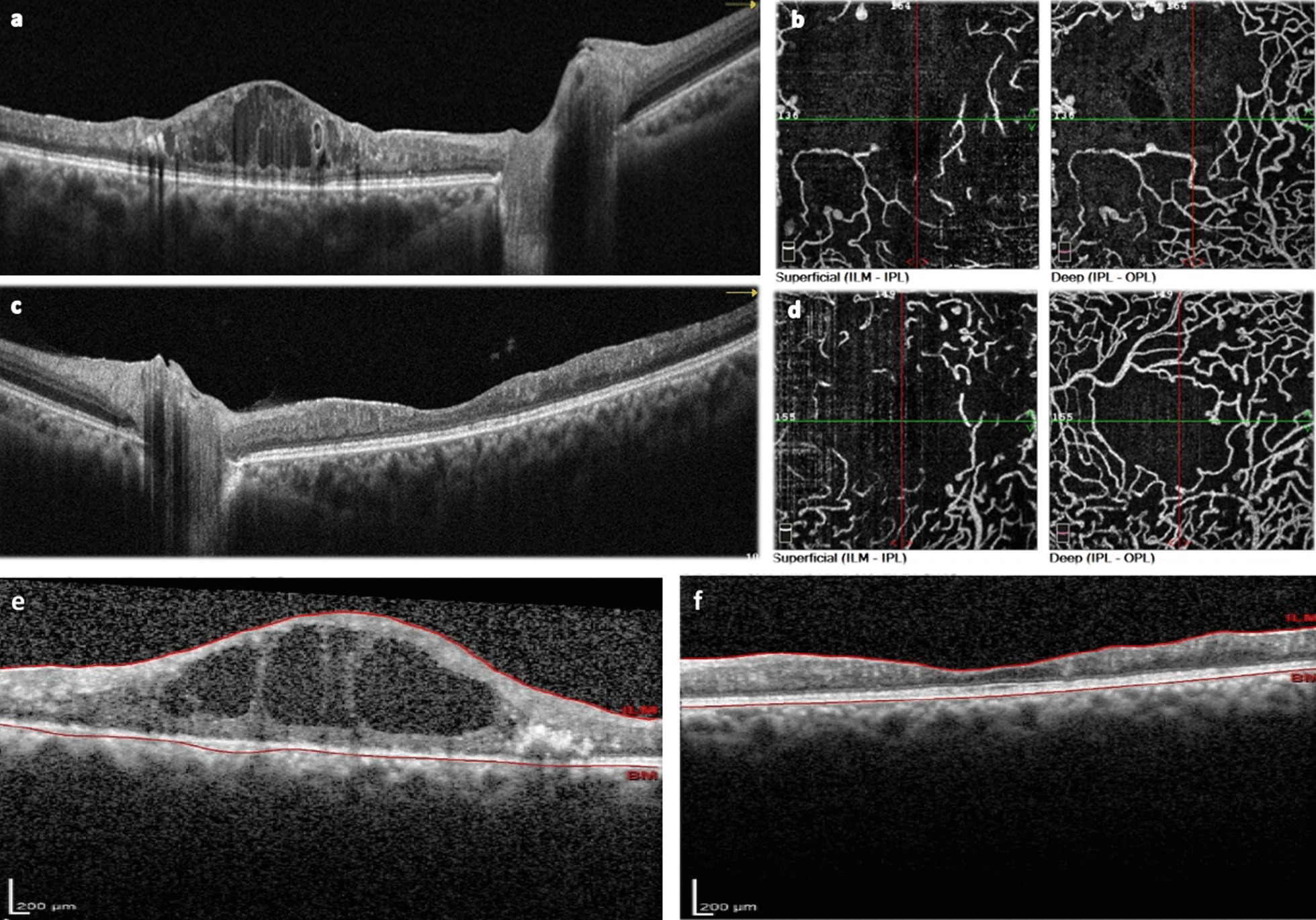
Macula OCT of the right eye (**a**) shows various-sized aneurysms within inner retinal layers, exudation, and retinal thickening in the form of CME. Macula OCT of the left eye (**c**) shows an atrophic thin retina with poorly defined layers. En face 3 × 3 mm OCTA in **b** and **d** demonstrates aneurysms and telangiectatic capillaries in deep and superficial capillary plexuses with sparse and distorted vessels in superficial plexus (left frames in **b**, **d**) and diffuse vascular rarefaction and dropout, telangiectatic vessels, and FAZ enlargement in deep capillary plexus (right frames in **b**, **d**), more prominent in the right eye (**b**). After 24 months, anti-VEGF injection in her right eye showed no improvement (**e**) and the left eye remained unchanged, without anti-VEGF injection (**f**)

A 58-year-old Iranian woman presented with gradual decline in vision in both eyes since adolescence that had worsened recently. Her past medical history was unremarkable and she neither smoked nor drank alcohol, nor she did not take any medication except intravitreal injection of anti-VEGF in her left eye 2 years previously. At presentation, her vital signs were stable (pulse rate 80 beats/minute, blood pressure: 115/60 mmHg, temperature: 36.9 °C), and baseline best-corrected visual acuity (BCVA) was 3/10 in both eyes. Anterior segment examination using slit-lamp biomicroscopy was unremarkable, and intraocular pressure was within normal limits. Dilated fundus examination revealed parafoveal telangiectatic vessels with capillary dilatation, as well as barely visible sclerotic vessels temporal to the macula, in both eyes. There was also dense circinate exudation and edema in the macula of the right eye. In the left eye, irregular perifoveal graying discoloration was evident (Fig. 1). Optical coherence tomography (OCT) of the macula in the right eye demonstrated aneurysms of various sizes located within inner retinal layers as well as exudation and retinal thickening. Retina in the macular area of the left eye was atrophic with disorganization of retina inner layers (DRIL). Of note, despite extensive capillary dropout, the architecture of the photoreceptors remained intact (Fig. 2a, c).

The 3 × 3 mm en face OCT angiography (OCTA) visualized aneurysms and telangiectasia in capillaries of deep and superficial capillary plexuses of the retina. In addition, vessels in the superficial plexus were sparse, distorted, and elongated with decreased density. More affected, deep capillary plexus showed diffuse rarefaction, telangiectasia, blunting of capillaries, and enlargement of the foveal avascular zone (FAZ) and perifoveal capillary loss, which were more prominent in the right eye (Fig. 2b, d).

**Fig. 3:**
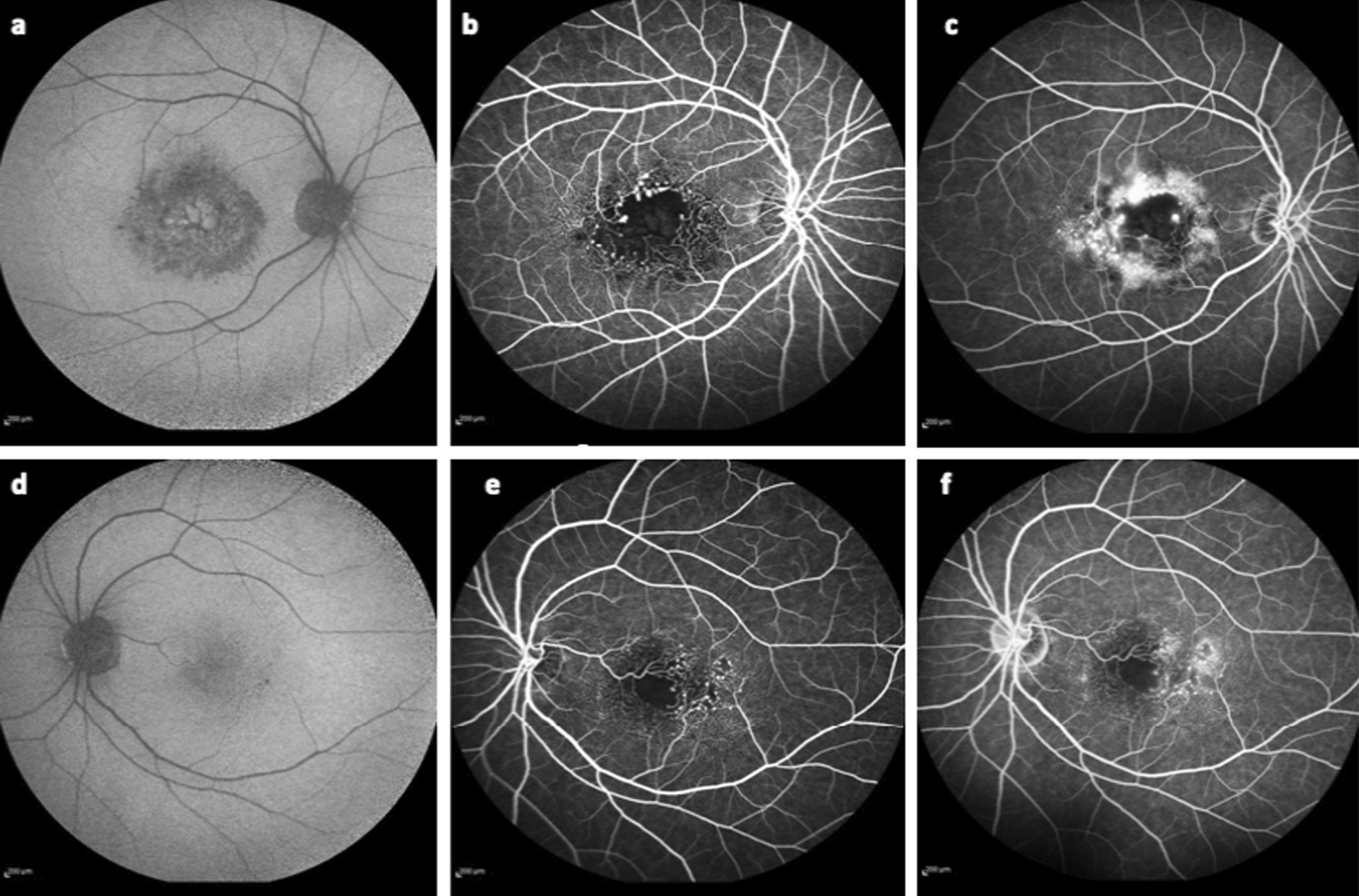
Fundus autofluorescence of right (**a**) and left (**d**) eye demonstrate florid-like hyper-autofluorescence in the right eye (**a**), suggesting CME. Fluorescein angiography of the right (**b**, **c**) and left (**e**, **f**) eye visualized numerous aneurysms that pool fluorescein, telangiectasia in the parafoveal area, enlarged FAZ, as well as small area with perifoveal capillary dropout, more prominent in the right eye (**b**, **c**)

Right eye fundus autofluorescence imaging showed florid-like hyper-autofluorescence, suggesting CME (Fig. 3a, d). Fluorescein angiography (FA) revealed dilated capillary bed in the parafoveal area, bordering capillary-free zone in both eyes. Also, pooling in the aneurysms and late intraretinal staining in the region of telangiectasia occurred, being more prominent in the right eye. The FAZ was enlarged and regions of capillary dropout were observed in the perifoveal area of both eyes (Fig. 3b, c, e, f).

We requested a neurological consultation for our patient to search for neurological impairment such as stroke, vasculitis, or white matter lesion. The result was as follows: The patient is alert and oriented with normal speech. Sensory or motor deficit is absent. Muscle strength is 5/5 bilaterally. Deep tendon reflexes are brisk, 2+, and symmetric in limbs. Babinski reflex is negative, and examination cranial nerves reveals them to be intact. Cerebellar tests such as finger to nose and heel to shin is smooth without dysmetria. Coordination is intact measured by walk toe–heel. Gait is steady with normal base. According to the possible chances of associated neurological deficits, brain magnetic resonance imaging (MRI) was ordered and came back as unremarkable.

Extensive systemic workup was also undertaken to search for an underlying etiology. Laboratory investigations including coagulation tests, blood chemistries, renal and liver function tests, urinalysis, and microbiologic studies showed no abnormalities. The results are as follows:

Complete blood count: RBC: 5.22 (× 10^6^/microL), Hct: 38.2%, HGB: 12.2 g/dL, WBC: 7.8 (× 10^3^/microL), Plt: 290 (× 10^3^/microL); liver function test: SGPT: 21 (U/L), SGOT: 30 (U/L) ALP: 300 (U/L); renal function test: BUN: 8 mg/dL Cr: 0.95 mg/dL; urinalysis: color: yellow, clear with negative protein, ketone, bilirubin and nitrates, pH: 6.0, specific gravity: 1.015; FBS: 78 mg/dL. Serology testing result was as follows: TB (IGRA), *Toxoplasma* IgG and IgM (0.1 CLIA), HIV, VDRL, RPR as well as FTA-ABS, and CRP were all negative. Anti dsDNA (10 IU/ml), ANA, anti-neutrophilic cytoplasmic antibody (c-ANCA) and p-ANCA, lupus anticoagulant (0.95) and anticardiolipin (0.1 AEU), and serum complement (95 mg/dL) were all negative. Serum protein electrophoresis, serum cryoglobulin (115 mg/dL) was within normal range. ESR (30 mm/hr), Ca: 9.6 mg/dL, P: 4.1 mg/dL, PT: 13 seconds and PTT: 31 seconds were all unremarkable.

We suggested an anti-VEGF injection in her right eye with macular edema. The left eye with atrophic retina remained untreated. Injection of intravitreal bevacizumab was not effective for the right eye, with no improvement. Her last follow-up OCT is illustrated in Fig. 2e, which was taken 2 years after anti-VEGF injection. Informed consent was obtained from the patient to report her condition.

## Discussion and conclusions

We report a case of bilateral macular telangiectasia with capillary obliteration in an otherwise healthy middle-aged woman, which was compatible with the diagnosis of MacTel type 3. Although presentation in female sex with bilateral involvement has been reported in 6% of patients with MacTel type 1 [2], the presence of occluded vessels around the macula argues against this diagnosis. Moreover, the area of capillary nonperfusion in our case is more extensive than expected for MacTel type 1. Bilateral ischemic parafoveolar region bordered by occlusive telangiectasia was considered characteristic for MacTel type 3. This subclass of MacTel had been eliminated from the updated classification of MacTel by Yannuzi [3].

All patients reported primarily by Gass as MacTel type 3 had some other systemic conditions such as polycythemia, gouty arthritis, or hypoglycemia, most probably predisposing these patients to capillary obstruction [2, 3]. Hyperreflexia, and central nervous system lesions were also noted in a subset of patients with type 3 MacTel [2, 5]. Maruko, however, published a series in which both patients with type 3 MacTel were female and had bilateral presentation. Visual acuity was good (the worst eye had BCVA of 0.7), despite retinal atrophy in OCT. The only systemic association was controlled hypertension in both patients [4]. Congenital multisystemic disorders may also accompany occlusive telangiectasia. In a genetic disorder called cerebroretinal microangiopathy with calcifications and cysts (CRMCC), characterized by obstruction of small vessels in the brain, retina, and gastrointestinal system, the retinal manifestations resembled MacTel type 3 with telangiectasia, obliteration, and capillary dropout as well as cystoid macular edema in one of the eyes. However, this multisystemic congenital condition is highly symptomatic, which led to early clinical diagnosis [6]. One may hypothesize that, in the absence of systemic disorders, macular pathology may remain unnoticed. Of note, frequent systemic assessment of patients with features of MacTel 3 should be kept in mind to discover any associated systemic condition that may manifest later.

To explain asymmetric retinal involvement and unilateral exudation in cases of MacTel type 3, Gass *et al.* speculated that deformed capillaries may have sufficient function for many years until gradual endothelial injury decompensated vascular drainage function and subsequent serous exudation, and retinal edema appears in the form of CME. The study also noted that the main reason for visual loss in patients with MacTel type 3 is ischemia of macula and subsequent atrophy of juxtafoveolar retina, rather than exudation. However, he reported a relatively good visual acuity in his series despite having an enlarged FAZ [1]. Considering the progressive nature of the course of capillary obstruction and longstanding edema in the macular area, a subsequent gradual decrease in vision due to ellipsoid zone disruption is expected [1, 4, 7, 8].

We observed macular thinning and DRIL in OCT B-scans in the absence of exudation in the left eye, which can be attributed to occlusive vasculopathy and ischemia in the macular area. The DRIL length has been demonstrated to correlate positively with FAZ size in retinal vascular disease [9]. In our patient, OCTA also confirmed juxtafoveolar capillary dropout that affected both the superficial and deep capillary plexuses. Nevertheless, the architecture of the photoreceptors remained intact. Likewise, Seraly *et al.* also reported a case with extensive macular capillary dropout with intact ellipsoid zone in a patient with type 3 MacTel [5].

There is no clear evidence in the literature on the role of anti-VEGF in MacTel cases [10, 11]. In accordance with this, we did not observe resorption of macular edema with anti-VEGF injection.

We present a case with features mostly compatible with the diagnosis of MacTel type 3. In 36 months of follow-up, no systemic association could be found. We hope this case sheds more light on the heterogeneity of this disease.

## Data Availability

The documents used and reported in the current study are available for consultation upon reasonable request.

## Abbreviations

OCT: Optical coherence tomography
OCTA: Optical coherence tomography angiography
IMT: Idiopathic macular telangiectasia
MacTel: Macular telangiectasia
CME: Cystoid macular edema
VEGF: Vascular endothelial growth factor
DRIL: Disorganization of retina inner layers
FAZ: Foveal avascular zone
FA: Fluorescein angiography
BCVA: Best-corrected visual acuity
CRMCC: Cerebroretinal microangiopathy with calcifications and cysts
